# An application of ARIMA model for predicting total health expenditure in China from 1978-2022

**DOI:** 10.7189/jogh.10.010803

**Published:** 2020-06

**Authors:** Ang Zheng, Quan Fang, Yalan Zhu, Chunling Jiang, Feng Jin, Xin Wang

**Affiliations:** 1 Department of Breast Surgery, the First Affiliated Hospital of China Medical University, Shenyang, Liaoning Province, China; 2 College of the Humanities and Social Sciences, China Medical University, Shenyang, Liaoning Province, China; 3 College of Basic Medical Science, China Medical University, Shenyang, Liaoning Province, China

## Abstract

**Background:**

China's health financing system has changed from the government-led mode under the planned economy to the diversified mode under the market economy. Equity in health financing has been a national health priority. This study aimed to predict changes in total health expenditure (THE), government health expenditure (GHE), social health expenditure (SHE) and out-of-pocket health expenditure (OOP) in China from 2018 to 2022, and to provide a theoretical basis for health policy adjustment.

**Methods:**

Based on health expenditure date of time series from 1978-2017, R3.5.1 software was used to construct the Autoregressive Integrated Moving Average (ARIMA) model.

**Results:**

The model of THE, GHE, SHE and OOP are ARIMA (3.3.0), ARIMA (1.3.1), ARIMA (2.4.0), ARIMA (2.2.2). According to the simulation results, in 2022, China's THE is expected to reach 8473.00 billion Yuan, and the constituent ratios in GHE, SHE and OOP will be 25.49%, 51.25% and 23.26%, respectively. The proportion of THE to GDP will continuously increase from 2018-2022 at a reasonable pace, while THE itself will increase rapidly.

**Conclusions:**

China should take effective measures to control the excessive growth of THE, keep decreasing the OOP percentage, and improve the efficiency and fairness of the use of health funds.

The total health expenditure (THE) is the total amount of money consumed by the whole society within a certain period for medical and health services, such as disease control, medical treatment, rehabilitation, and health promotion, which occurred as a form of currency in a country or region [1,2]. It contains government health expenditure (GHE), social health expenditure (SHE) and out-of-pocket health expenditure (OOP) [3]. THE objectively reflects the relationship between national macro-health policies and economic development, which provides information for the government in making and adjusting related health policies. Equity in health financing is not only related to the amount of THE, but also the structure among the three components [4]. Increasing the proportion of OOP may cause people to forgo care and become poor [5-7]. In 2010, OOP pushed an estimated 97 million people (1.4% of the world’s population) below the US$ 1.90 per day extreme poverty line [8]. Increasing the proportion of GHE to SHE would more equally distribute health costs and reduce poverty rates [9].

China is considered to have one of the highest cash payment rates in Asia and irrational health financing structures is the main problem [10]. In 1998, 44.8% of urban citizens and 79.1% of rural residents in China were not covered by any social health security system and they paid for health care by OOP [11]. According to a report by the World Health Organization (WHO) in 2000, in terms of equity in health financing, China was listed as fourth from bottom among 192 member states, which was far from expectations [12]. Therefore, to solve this problem, a New Cooperative Medical Scheme (NCMS) and a new governmental public health service funding had been adopted in 2003 and 2009, respectively [13,14]. Since the reform of the medical and health system, 90% of China's population has been covered by medical insurance [15].

Although China's THE increased by 476 times from 1978 to 2017, the inflation and continued population growth may lead to the per capita THE lower. There was a slight downward trend in GHE and THE, while OOP increased slightly before 2003. After that, this phenomenon began to reverse. Besides, the Proportion of THE in GDP had increased year by year, accounting for 6.36% in 2017. Meanwhile, the proportion of GHE in THE fluctuated in recent years after a continuous increase, and OOP appeared a sluggish decline [16]. This makes health policy formulation and implementation more difficult.

Scientific analysis and prediction of THE and its composition can provide recommendations for evaluating and formulating health financing policies, health reform, and development strategies. Several previous studies focused on the change of THE, while the change trend and forecast of the financing source of THE was rarely reported [17-19]. Wang 's paper used Autoregressive integrated moving average (ARIMA) model to analyze and predict THE and its composition in China from 1978 to 2016 [20]. But it had two major problems. One was limited predictive power in this model, which made it more difficult to make policy and easier to provide wrong information. For example, GHE as a proportion of THE has decreased by 1.11%, while OOP has risen by 0.02% in 2017, which is contrary to Wang’s predictions [20]. Another was unreliable THE caused by errors accumulation. THE was calculated by the predicted GHE, SHE, OOP, but not by ARIMA model. To avoid the error above and get precise information, this study analyzed the changing trend of THE and the changing characteristics of financing sources from the reform and opening up to 2017, and to predict the change trend of China's total THE from 2018-2022 using the ARIMA model time series method, from three sources: GHE, THE, and OOP.

## METHODS

### Data source

The paper cited the THE, CHE, SHE, OOP in China from 1978 to 2017, from the China Statistical Yearbook [16].

### Model construction

The ARIMA model has the advantages of simplicity and stability. THE is driven by several factors and the ARIMA model is considered the most appropriate model under existing conditions [21]. (ARIMA) (p, d, q) model is an extension of autoregressive (AR), moving average (MA), and ARMA model [22]. p is the order of AR; d is the degree of difference and q is the order of MA. ARIMA model is developed with four synergistic steps including time series stationary, model identification, parameter estimation and diagnostic checking [23]. First, THE, CHE, SHE, OOP were plotted against time to detect and correct for non-stationarity of the time series. Stationarity was judged by the Augmented Dickey-Fuller (ADF) test. Probability values less than 0.05 were considered statistically significant, indicating that the sequence was stationary. Second, identified AR and MA were needed by calculating the autocorrelation (ACF) and partial autocorrelation (PACF) functions. Next, models of different orders were fitted and compared with Akaike information criteria (AIC) to evaluate the improvement of fitting [24]. Auto Arima function package in R software can quickly find the most suitable model. Last, temporal autocorrelation was confirmed to have been no longer present in model residuals using the Ljung-Box test (*P* > 0.05) [25]. R statistical software version 3.5.1 (R Foundation,Vienna, Austria) was used to carry out the analyses.

### Model prediction tests

THE, SHE, GHE and OOP from 2013 to 2022 were predicted according to ARIMA models. The validity of the model was evaluated by comparing the difference between the predicted value and the real value in 2013-2017 and the MAPE (Mean absolute percent error) in 2017-2022.

## RESULTS

### Current THE in China

Since 1978, both China's GDP and THE have been increasing year by year. The ratio of THE to GDP increased from 3.00% in 1978 to 6.36% in 2017. GHE, SHE, OOP also increased year by year. Among them, GHE increased from 32.16% in 1978 to 38.69% in 1986. After that, GHE decreased continuously. SHE had a similar trend to GHE. OOP has dropped below 30% in recent years from a high of 59.97% in 2001 (**Table 1**).

**Table 1:** China’s GDP and total health financing composition*

Years	GDP (billion Yuan)	THE		GHE		SHE		OOP	
		**Absolute number (billion Yuan)**	**Proportion in GDP (%)**	**Absolute number (billion Yuan)**	**Proportion in THE (%)**	**Absolute number (billion Yuan)**	**Proportion in THE (%)**	**Absolute number (billion Yuan)**	**Proportion in THE (%)**
1978	367.87	11.02	3.00	3.54	32.16	5.23	47.41	2.25	20.43
1979	410.05	12.62	3.08	4.06	32.21	5.99	47.45	2.57	20.34
1980	458.76	14.32	3.12	5.19	36.24	6.10	42.57	3.04	21.19
1981	493.58	16.01	3.24	5.97	37.27	6.24	38.99	3.80	23.74
1982	537.34	17.75	3.30	6.90	38.86	7.01	39.49	3.84	21.65
1983	602.09	20.74	3.44	7.76	37.43	6.46	31.12	6.52	31.45
1984	727.85	24.21	3.33	8.96	36.96	7.36	30.41	7.90	32.64
1985	909.89	27.90	3.07	10.77	38.58	9.20	32.96	7.94	28.46
1986	1037.62	31.59	3.04	12.22	38.69	11.04	34.93	8.33	26.37
1987	1217.46	37.96	3.12	12.73	33.53	13.73	36.16	11.51	30.31
1988	1518.04	48.80	3.21	14.54	29.79	19.00	38.93	15.27	31.28
1989	1717.97	61.55	3.58	16.78	27.27	23.78	38.64	20.98	34.09
1990	1887.29	74.74	3.96	18.73	25.06	29.31	39.22	26.70	35.73
1991	2200.56	89.35	4.06	20.41	22.84	35.44	39.67	33.50	37.50
1992	2719.45	109.69	4.03	22.86	20.84	43.16	39.34	43.67	39.81
1993	3567.32	137.78	3.86	27.21	19.75	52.48	38.09	58.10	42.17
1994	4863.75	176.12	3.62	34.23	19.43	64.49	36.62	77.41	43.95
1995	6133.99	215.51	3.51	38.73	17.97	76.78	35.63	100.00	46.40
1996	7181.36	270.94	3.77	46.16	17.04	87.57	32.32	137.22	50.64
1997	7971.50	319.67	4.01	52.36	16.38	98.41	30.78	168.91	52.84
1998	8519.55	367.87	4.32	59.01	16.04	107.10	29.11	201.76	54.85
1999	9056.44	404.75	4.47	64.10	15.84	114.60	28.31	226.06	55.85
2000	10 028.01	458.66	4.57	70.95	15.47	117.19	25.55	270.62	58.98
2001	11 086.31	502.59	4.53	80.06	15.93	121.14	24.10	301.39	59.97
2002	12 171.74	579.00	4.76	90.85	15.69	153.94	26.59	334.21	57.72
2003	13 742.20	658.41	4.79	111.69	16.96	178.85	27.16	367.87	55.87
2004	16 184.02	759.03	4.69	129.36	17.04	222.54	29.32	407.14	53.64
2005	18 731.89	865.99	4.62	155.25	17.93	258.64	29.87	452.10	52.21
2006	21 943.85	984.33	4.49	177.89	18.07	321.09	32.62	485.36	49.31
2007	27 023.23	1157.40	4.28	258.16	22.31	389.37	33.64	509.87	44.05
2008	31 951.55	1453.54	4.55	359.39	24.73	506.56	34.85	587.59	40.42
2009	34 908.14	1754.19	5.03	481.63	27.46	615.45	35.08	657.12	37.46
2010	41 303.03	1998.04	4.84	573.25	28.69	719.66	36.02	705.13	35.29
2011	48 930.06	2434.59	4.98	746.42	30.66	841.65	34.57	846.53	34.77
2012	54 036.74	2811.90	5.20	843.20	29.99	1003.07	35.67	965.63	34.34
2013	59 524.44	3166.90	5.32	954.58	30.14	1139.38	35.98	1072.93	33.88
2014	64 397.40	3531.24	5.48	1057.92	29.96	1343.78	38.05	1129.54	31.99
2015	68 905.21	4097.46	5.95	1247.53	30.45	1650.67	40.29	1199.27	29.27
2016	74 358.55	4634.49	6.23	1391.03	30.01	1909.67	41.21	1333.79	28.78
2017	82 712.20	5259.83	6.36	1520.59	28.90	2225.88	42.30	1513.36	28.80

GHE – government health expenditure, THE – total health expenditure, OOP – out-of-pocket health expenditure, GDP – gross domestic product; SHE – social health expenditure

*Source: Authors’ analysis and China Statistical Yearbook (1979-2018).

### Model estimation and checking

THE, GHE, SHE and OOP are non-stationary time series, which need to be differentiated. Under the condition of ADF<0.05, d_THE_ = 3, d_GHE_ = 3, d_SHE_ = 4, d_THE_ = 2, Draw the corresponding ACF and PACF. The possible model is determined according to ACF, PACF and the Auto Arima function package in R software. If *P* value of residual LB test is greater than 0.05, the minimum value of AIC means that the model is optimal. Finally, the model of THE, GHE, SHE and OOP are ARIMA (3.3.0), ARIMA (1.3.1), ARIMA (2.4.0), ARIMA (2.2.2).

### Forecasting results

The predicted values were calculated according to the respective ARIMA model. Compared with real values, the mean relative error of GHE, THE, SHE and OOP in 2013-2017 were 1.36%, 2.76%, 2.33%, and 2.01%, respectively (**Table 2**). In 2018-2022, the predicted values of THE, GHE, SHE and OOP all keep rising, and an absolute value of increase keeps increasing. In 2022, THE, GHE, SHE and OOP were 9447.90 billion Yuan, 2260.50 billion Yuan, 4544.31 billion Yuan, and 2062.42 billion Yuan, respectively. GHE and OOP's share of THE continued to decrease, from 28.02% to 25.49% and 27.97% to 23.26%, respectively, whereas SHE increased from 44.01% to 51.25%. The lowest MAPE was THE (2.57%), followed by SHE (3.73%), GHE (4.20%), and OOP (6.07%). The specific situation of forecast every year was presented in **Table 3****.**

**Table 2:** Comparison of predicted and actual values from 2013 to 2017

Category	Years	Predictive value	Real value	Absolute error	Relative error (%)	Mean relative error (%)
**THE**	2013	3212.36	3166.90	45.46	1.44	1.36
	2014	3548.25	3531.24	17.01	0.48	
	2015	4005.31	4097.46	92.16	2.25	
	2016	4570.95	4634.49	63.54	1.37	
	2017	5194.56	5259.83	65.27	1.24	
**GHE**	2013	983.43	954.58	28.85	3.02	2.72
	2014	1064.27	1057.92	6.35	0.60	
	2015	1170.56	1247.53	76.97	6.17	
	2016	1403.60	1391.03	12.57	0.90	
	2017	1564.47	1520.59	43.88	2.89	
**SHE**	2013	1175.87	1139.38	36.49	3.20	2.33
	2014	1313.36	1343.78	30.42	2.26	
	2015	1573.01	1650.67	77.67	4.71	
	2016	1925.85	1909.67	16.18	0.85	
	2017	2211.28	2225.88	14.60	0.66	
**OOP**	2013	1058.06	1072.93	14.87	1.39	2.01
	2014	1170.71	1129.54	41.17	3.65	
	2015	1216.32	1199.27	17.05	1.42	
	2016	1313.32	1333.79	20.47	1.53	
	2017	1481.81	1513.36	31.55	2.08	

GHE – government health expenditure, THE – total health expenditure, OOP – out-of-pocket health expenditure; SHE – social health expenditure

**Table 3:** The forecast of China's THE and financing composition from 2018 to 2022

Category	Years	Predictive value	95% CI	Proportion in THE (%)	MAPE (%)
**THE**	2018	5942.01	5822.97-6061.05	-	2.57
	2019	6756.98	6513.70-7000.26	-	
	2020	7562.75	7154.31-7971.19	-	
	2021	8473.00	7843.70-9102.30	-	
	2022	9447.90	8506.60-10 389.21	-	
**GHE**	2018	1662.91	1595.73-1730.09	28.02	4.20
	2019	1805.07	1680.88-1929.27	27.43	
	2020	1953.33	1751.72-2154.94	26.91	
	2021	2104.65	1815.35-2393.95	26.24	
	2022	2260.50	1870.55-2650.45	25.49	
**SHE**	2018	2611.44	2554.52-2668.35	44.01	3.73
	2019	3013.57	2908.65-3118.49	45.80	
	2020	3464.06	3308.89-3619.24	47.72	
	2021	3980.31	3750.33-4210.28	49.63	
	2022	4544.31	4220.75-4867.87	51.25	
**OOP**	2018	1659.64	1601.17-1718.12	27.97	6.07
	2019	1761.22	1651.45-1871.00	26.77	
	2020	1841.49	1688.68-1994.31	25.37	
	2021	1935.55	1743.32-2127.79	24.13	
	2022	2062.42	1824.00-2300.84	23.26	

GHE – government health expenditure, THE – total health expenditure, OOP – out-of-pocket health expenditure, SHE – social health expenditure, MAPE – mean absolute percentage error

## DISCUSSION

China's health financing system has changed from the government-led mode under the planned economy to the diversified mode under the market economy [26]. From 1978 to 2017, China had achieved remarkable results in total health financing and financing structure, but now there is still space for more optimized and efficient improvement. At present, China’s health care costs have grown much faster than economic growth by far [27]. According to the requirements of “Healthy China 2020” Strategic Planning, the proportion of THE in GDP will reach 6.5% to 7.0% [28]. So we will focus on the optimization of financing structure. Irrational health financing structures directly lead to a severe disease economic burden on the population and even harm the socio-economic situation in the short or long term [29].

The mean average error of the predicted value compared with the real value from 2013 to 2017 in the Arima model adopted in this study was less than 3%, and the highest MAPE value was only 6.07% for the predicted results from 2018 to 2022, while the others were less than 5.00%. Because the fitting degree is better than other studies [19], the prediction results are greater value for reference.

China’s THE is increasing exponentially [30]. The reasons may be the increasing financial input to medical institutions, the policy of full coverage of medical insurance, the acceleration of the aging trend of China’s population and the increasing demand of the people for a better life. In 2022, the proportion of THE in GDP will be approximately 8.2% (the average growth rate, as measured by GDP growth in 2017, maybe slightly lower). This indicates that the total funding of financing for health in China is continuously improving, and the health industry is constantly advancing. In the United States, THE accounted for 17.1% of GDP in 2014. However, if China keeps the trend of rapid rise, it may push up the fiscal deficit and debt level and increase the risk of a national debt crisis, which may also lead to increased government taxes. High taxes are bound to curb the potential of economic growth [31], which is bound to adversely affect the sustainability of health financing. That’s not going to happen in 2022, because we’re going to step up our efficiency priorities to be better able to deal with it when it does happen.

### GHE for THE

Nationwide, although GHE has been increasing continuously since 2015, the proportion of GHE has been decreasing due to the rapid growth of SHE. GHE is predicted to fall to 25.49% in 2022, decreased by 3.41%. Compared with developed countries, the proportion of GHE was far less than the United States (43.2% in 2013) and Japan, Canada and Italy (all above 70%) [32,33]. Under the premise of certain GHE, to achieve higher output effect and maximize benefits, more attention should be paid to the rational allocation and use efficiency of funds. Besides, the guiding role of the government in the field of health should continue to be emphasized. We should improve the construction of the medical security system, expand the coverage of serious disease insurance and increase its reimbursement rate, and include safe and effective medicines in the national list of medicines for basic medical insurance, so as to reduce the burden of medical expenses on individuals.

### SHE for THE

In 2001, the ratio of SHE was only 24.1% in China, which was the lowest in all years. In the 15 years before 2017, the ratio of SHE increased year by year except in 2011, which might be related to universal health care. The population coverage by health insurance was from around 29.7% in 2003 to over 90% at the end of 2010 [27]. In the forecast results, SHE is the fastest growing and the largest increment. The proportion of SHE in THE will be 51.25%. SHE mainly consists of two parts: the basic medical insurance fund and the social capital input paid by the units and individuals, which are included in the coverage of urban employees' basic medical insurance [34]. In the case of limited GHE, SHE should be increased to alleviate the problem of “expensive medical treatment”. We can increase the contribution of enterprises and individuals, encourage social capital to set up hospitals, especially traditional Chinese medicine. At the same time, it is necessary to increase the social health input, give play to the advantages of “simplicity, convenience, testing and integrity” of traditional Chinese medicine, meet people's diversified demands for medical services. All of these can be used to effectively control the unreasonable growth of health expenditure [35].

### OOP for THE

In developing countries, such as China, OOP forms a greater proportion of the sources of health care financing [36]. Although the proportion of OOP has been decreasing worldwide, the decline in China is more pronounced [8]. Unlike GHE, the proportion of OOP in THE keeps going down, reaching to 23.26% in 2022, which is in line with the test requirements of the world health organization in the Asia-pacific health financing strategy (2010-2015) in 2009. The proportion of the total personal cash health expenditure will not exceed 30% to 40% [3]. But WHO illustrated that when individual cash health spending falls to 15%-20%, the chance of economic hardship and poverty drops to negligible levels [37].

In China, high-quality medical resources are mostly concentrated in the 3A (Class Three/Grade A) hospitals. Drug zero markup policy implementation lead to increase the hospital checking cost, medical service cost prices and the cost of the entire health system, so the actual OOP of patients remain very high [38]. Besides implementing policies, the key is to transfer the burden of individual medical treatment. Therefore, it is crucial to ensure the source of social funds.

The international significance of this study can be discussed in the following aspects: First, this study used the ARIMA model time series method, from three sources: GHE, THE, and OOP. This innovative model can be applied in other countries. Second, although this study was analyzed based on data from China, it can be used by other countries for comparison. Third, other countries can adjust their own policies according to the policy in China and the suggestions in this paper. High health expenditure and irrational health financing structures is an issue facing the whole world, and the rise of which not only relies on different medical system respectively, but also correlates with the common aspects. The changing trends of THE and financing sources in China is an integrated part of health accounting internationalization study. The prediction of China's total THE provide reference for the study around the world in the future.

This study has some limitations. First, ignoring inflation will lead to absolute value estimation less accurate, so this study adopts ratio. Second, the model's estimates are based on data from the past, and it will still be tested given the uncertainties of the future. Third, the premise of the forecast is that the existing policies and statistical caliber do not change significantly. Otherwise, the predicted results are meaningless.

## CONCLUSIONS

From 2018-2022, THE will increase rapidly. The proportion of GHE, OOP in THE will decline, and SHE will grow fastest. The change of health financing structure in China will slow down. In the next step, China should take effective measures to control the rapid growth of the total health expenditure, increase social health input continuously, improve the efficiency and fairness of the government's health funds, and focus on the transformation of patient-centered treatment and prevention.
